# Vagus nerve stimulation reduces spreading depolarization burden and cortical infarct volume in a rat model of stroke

**DOI:** 10.1371/journal.pone.0236444

**Published:** 2020-07-23

**Authors:** Jan Lindemann, Cordula Rakers, Hana Matuskova, Bruce J. Simon, Thomas Kinfe, Gabor C. Petzold

**Affiliations:** 1 German Center for Neurodegenerative Diseases (DZNE), Bonn, Germany; 2 electroCore Inc., Basking Ridge, New Jersey, United States of America; 3 Department of Neurosurgery, University Hospital Bonn, Bonn, Germany; 4 Division of Functional and Stereotactic Neurosurgery, Department of Neurosurgery, University Hospital Erlangen, Erlangen, Germany; 5 Division of Vascular Neurology, Department of Neurology, University Hospital Bonn, Bonn, Germany; University of Warwick, UNITED KINGDOM

## Abstract

Cortical spreading depolarization (SD) waves negatively affect neuronal survival and outcome after ischemic stroke. We here aimed to investigate the effects of vagus nerve stimulation (VNS) on SDs in a rat model of focal ischemia. To this end, we delivered non-invasive VNS (nVNS) or invasive VNS (iVNS) during permanent middle cerebral artery occlusion (MCAO), and found that both interventions significantly reduced the frequency of SDs in the cortical peri-infarct area compared to sham VNS, without affecting relative blood flow changes, blood pressure, heart rate or breathing rate. In separate groups of rats subjected to transient MCAO, we found that cortical stroke volume was reduced 72 h after transient MCAO, whereas stroke volume in the basal ganglia remained unchanged. In rats treated with nVNS, motor outcome was improved 2 days after transient MCAO, but was similar to sham VNS animals 3 days after ischemia. We postulate that VNS may be a safe and efficient intervention to reduce the clinical burden of SD waves in stroke and other conditions.

## Introduction

Despite great therapeutic advances in recent years, ischemic stroke remains a major cause of death and disability worldwide, mainly because most therapeutic options are limited to the first hours after stroke onset and are usually performed by highly specialized comprehensive stroke centers. Hence, there is a clear need to develop novel adjunctive treatment strategies that can be applied easily and do not interfere with established therapeutic protocols such as thrombolysis and thrombectomy. One such potential intervention is cervical invasive or non-invasive vagus nerve stimulation (VNS), which is an FDA-approved and clinically widely used adjunctive therapy for partial epilepsy, drug-resistant depression and primary headache disorders. In addition, VNS has also shown beneficial effects on stroke volume and outcome in different animal models of focal ischemia [1–6]. The effects on migraine appear to be partially related to the ability of VNS to decrease the susceptibility for cortical spreading depolarization (SD) waves, which induce spreading depression of activity, the assumed electrophysiological correlate of migraine aura [7]. These depolarization waves, which are defined by a sudden and sustained mass depolarization in the gray matter, can occur across a spectrum or continuum of brain states–from healthy brain tissue, as in the migraine aura, to different parts of an ischemic area, such as the severely energy-depleted infarct core as well as the mildly ischemic tissue surrounding it [8]. In the course of acute stroke, SDs are induced by failure of the sodium pump in the wake of ischemia, and they induce cytotoxic edema, impose a strong metabolic burden on the ischemic tissue and can further decrease cerebral blood flow (CBF) through inverse coupling [9–12]. Moreover, they are assumed to contribute in multiple ways to the development of the ionic and vasogenic edema at later stages of ischemia [10,13,14].

Hence, SDs are among the most important contributors to infarct generation, cell death and injury expansion in experimental models and clinical cases of stroke as well as other acute neurological disorders [8,15]. Importantly, however, the consequences of VNS for the pathophysiological correlate of these SD waves in the context of acute ischemic stroke have remained unexplored. Hence, given the translational and clinical importance of SDs for stroke outcome, we here aimed to determine whether VNS may represent a novel non-pharmacological approach to inhibit SDs in a rodent model of focal ischemia.

## Materials and methods

### Animals

We used adult male Wistar rats (9–11 weeks; 340–430 g, Charles River (Sulzfeld, Germany) and Janvier Labs (Saint-Berthevin Cedex, France)). Animals were maintained in a pathogen-free and climate-controlled environment with access to water and food *ad libitum*. All experiments were performed in accordance with institutional animal care guidelines and approved by the governmental animal welfare committee of the *Landesamt für Natur*, *Umwelt und Verbraucherschutz NRW*. The experiments are reported in compliance with the ARRIVE guidelines and were carried out in accordance with the EU Directive 2010/63/EU for animal experiments.

### Cranial window preparation and middle cerebral artery occlusion (MCAO)

Anesthesia was induced by inhalation of 3% isoflurane (Abbott) through a face mask and was maintained at 2.0–2.5% isoflurane during surgery and at 1.0–1.5% during experiments. Body temperature was kept at 37 ± 0.5°C using a controlled heating plate.

Buprenorphine (Reckitt Benckiser; 0.05 μg/g) was administered intraperitoneally before surgery for analgesia, and lidocaine (2%, with 0.001% adrenalin; AstraZeneca) was injected subcutaneously before cranial window preparation. After fixation in a stereotaxic frame, the scalp was removed and a craniotomy (diameter, 4 mm) was created over the left somatosensory cortex (centered 4 mm lateral and 3 mm posterior to bregma) using a dental drill. During MCAO, the window was temporarily covered by a coverslip (diameter, 6mm; VWR) fixed with silicone elastomer (WPI).

MCAO was performed as previously described [12,16]. Briefly, the animal was placed on its back and an incision was made above the thyroid gland. The left common carotid artery was separated and ligated with a suture proximal to the bifurcation. The external carotid artery was ligated by a second ligation distal to the bifurcation. A vascular clamp (Fine Science Tools) was applied around the internal carotid artery. Subsequently, a small incision was made in the common carotid artery and a filament was inserted for permanent MCAO (pMCAO) induction (3–4 mm coating length, 0.35 ± 0.02 mm tip diameter; Doccol). After removal of the clip, the filament was pushed forward until it occluded the middle cerebral artery, and was fixed in place with a suture. For transient MCAO (tMCAO), the filament was removed after 60 min. MCAO control animals received a sham surgery. Buprenorphine (0.05 μg/g) was administered intraperitoneally for 48 h after surgery for analgesia.

### Electrophysiology

After pMCAO induction, the anesthetized animal was fixed in a stereotaxic frame. Elastomer and coverslip were removed, the dura was removed, and a borosilicate glass microelectrode (tip diameter, 2–5 μm; WPI) connected to a head stage (EXT-02; NPI Electronic) was inserted 100–200 μm below the pial surface from an oblique angle. A reference electrode (diameter, 4 mm; Warner Instruments) was placed subcutaneously at the neck. The cranial window was kept moist by a drop of mineral oil (HP50.2; Carl Roth). The DC potential was amplified (EXT-02F/2; NPI Electronic), digitized (micro1401; CED) and analyzed offline using Spike2 (CED).

### Cerebral blood flow measurements

During pMCAO, relative cerebral blood flow (CBF) changes were measured by laser speckle contrast imaging (PeriCam PSI; Perimed) using PimSoft software (Perimed) at a resolution of 60 μm/pixel. Baseline CBF was recorded for 5 min before pMCAO induction. During tMCAO, local CBF was monitored by laser Doppler flowmetry (VMS LDF2; Moor instruments) through the thinned skull.

### Vagus nerve stimulation

Invasive vagus nerve stimulation (iVNS) was performed according to previous protocols [1] with minor modifications. A nerve cuff electrode (100 μm platinum contacts, 1.5 mm inner cuff diameter; Microprobes) was placed around the left vagus nerve and sutured at this position. 30 min after MCAO induction, 0.3 ms pulses of a 0.5 mA constant current were delivered through an externalized lead at 25 Hz for 30 s, and this electrical stimulation was repeated every 5 min for 1 h, using a Master-8 stimulator (A.M.P.I., Israel). In control animals that received a sham VNS procedure, a silicone tube of 1.5 mm inner diameter was implanted instead of the electrodes and no stimulus was applied.

Non-invasive vagus nerve stimulation (nVNS) was administered according to previous protocols [3]. Briefly, the vagus nerve was stimulated transcutaneously by an external transcutaneous stimulator (electroCore Inc.). For stimulation, the neck was shaved, contact gel (electroCore Inc.) was applied to the skin, and the stimulator was attached to the neck of the animal above the left vagus nerve. 30 min after MCAO, stimulation (1-ms pulses of 5 kHz sine waves repeated at 25 Hz) was applied for 2 min. This stimulation was repeated once after 15 min. In control animals that received a sham VNS procedure, the stimulator was attached to the neck without triggering the device.

We stimulated the left vagal nerve for better comparability with the majority of previous preclinical and clinical studies that also stimulated the left vagus nerve to minimize cardiac side effects [6], although later clinical studies have shown that noninvasive clinical devices can safely be used on both sides. Moreover, previous studies have shown that the effects on SDs or MCAO outcome are similar with ipsilateral or contralateral stimulation [3,7].

### Pulsoxymetry and blood pressure measurement

Heart rate, respiratiory rate, and oxygen saturation were monitored in all animals by pulse oximetry at the right hindpaw (MouseOx Plus, STARR Life Science Corp.). During pMCAO, blood pressure was measured using a non-invasive tail cuff system (CODA Monitor, Kent Scientific Corp.).

### Behavioral tests

The Garcia neuroscore is a composite neurological score particularly suitable for assessing neurological deficits after tMCAO [17]. Different modalities of the sensorimotor system (spontaneous activity, symmetrical posture of extremities, forelimb extension, climbing, body perception, vibrissae testing) are tested, resulting in a score between 3 points (severe impairment) and 18 (no deficit).

The *g*rid walk test assesses foot placement deficits during locomotion. Healthy animals can exactly place their paws on the grid, while paretic limbs of animals after ischemia can slip through the grid (= foot fault) [18]. The animal was placed on an elevated wire mesh (50x50x50 cm, 3 cm squares), and the first 50 forepaw steps and 30 hind paw steps were evaluated. The difference of the number of ipsilateral foot faults and contralateral foot faults was divided by the total number of steps for each trial (foot fault index) [19].

Both tests were performed before tMCAO. The Garcia score was assessed 1, 2, and 3 days after surgery, and the grid walk test was performed on days 2 and 3.

### Infarct volumetry

2 mm brain sections were taken from saline-perfused brains using a rat brain matrix (WPI) and stained with 2, 3, 5-triphenyltetrazolium chloride (TTC; Sigma) at 37°C for 15 min. Infarct volume was calculated by numerically integrating lesion areas after edema correction [20] using ImageJ (version 1.47v, NIH).

### Statistics

All animals were randomized, and assessment was performed by investigators blinded to the group assignment. Animals were only included when successful MCAO was verified by CBF measurements and surgical window preparation was successful. Animals were excluded when they died or had to be euthanized before reaching the primary end point. We used the Mann-Whitney test or unpaired t-test for comparisons between two groups and the two-way repeated measures ANOVA and Dunnett’s multiple comparisons test for multiple measurements in the same groups. Data were analyzed using Prism 8 (GraphPad) and are expressed as Tukey’s box-and-whisker plots indicating the median, mean, interquartile range (IQR), and 1.5 IQR. A value of p <0.05 was accepted as statistically significant.

## Results

### Invasive and non-invasive VNS reduce SD frequency after focal ischemia

**Fig 1:**
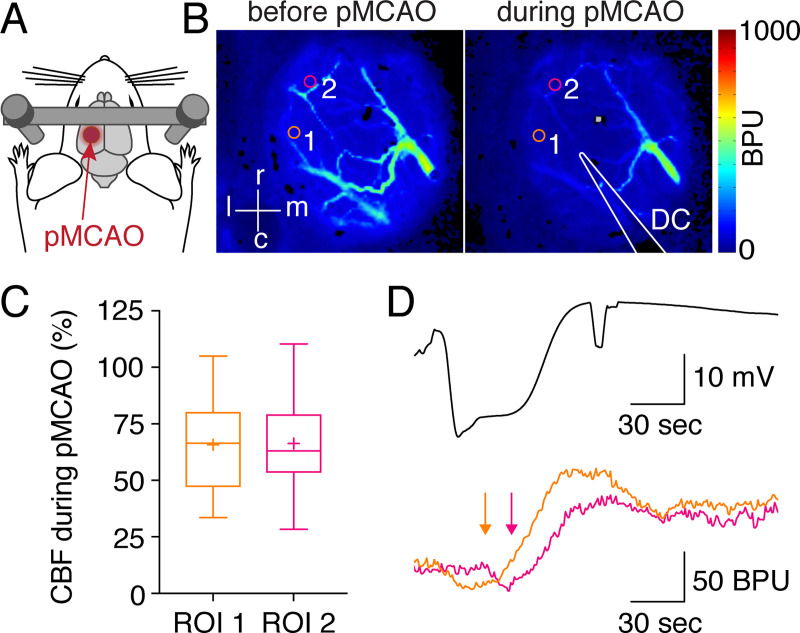
Measuring SDs in a rat model of focal stroke. (A-B) Permanent middle cerebral artery occlusion (pMCAO) was induced in rats, and relative changes in cerebral blood flow (CBF) in peri-infarct cortex were measured through a cortical window using laser speckle contrast imaging (BPU, blood perfusion units; l, lateral; m, medial; r, rostral; c, caudal). Regions of interest (ROI) are depicted by circles. An intracortical electrode was inserted for direct current (DC) potential measurement. (C) CBF relative to baseline decreased in both ROIs following pMCAO induction (n = 32 animals), indicating that experiments were carried out in peri-infarct cortex. (D) Representative examples of an SD after MCAO. A transient negative deflection of the DC potential, measured using an intracortical electrode, is accompanied by propagating transient CBF changes (arrows show the delay of onset indicating propagation).

To determine the effects of VNS on SDs in a stroke model, we induced focal ischemia by permanent MCAO (pMCAO) in rats, as this model is characterized by a high rate of SDs during the vessel occlusion time [12]. By measuring relative changes in cortical CBF using laser speckle contrast imaging through cranial window preparations, we confirmed that the area of interest was located in peri-infarct cortex (Fig 1A–1C). Simultaneously, we measured the direct current (DC) potential during pMCAO by placing an intracortical electrode in the peri-infarct area. SDs in that area were identified by the typical negative DC-potential shift followed by a propagating CBF response triggered by the SDs (Fig 1D).

**Fig 2:**
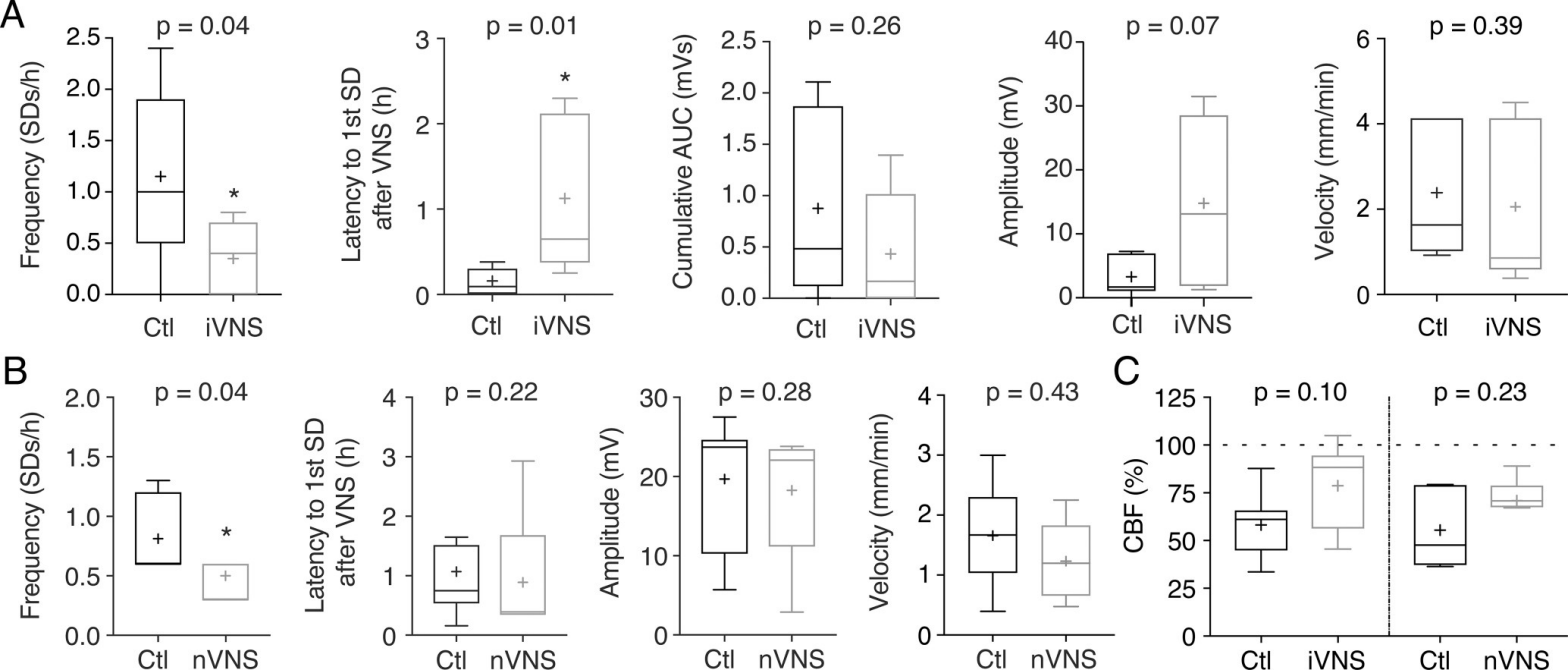
nVNS and iVNS reduce SD frequency during focal ischemia. (A) iVNS, administered 30 min after MCAO for 60 min, strongly reduced SD frequency and increased the latency to the first SD after iVNS initiation, whereas the cumulative area under the curve (AUC) as well as amplitude and SD velocity remained unchanged (n = 8 rats undergoing iVNS vs. n = 8 rats undergoing a sham VNS procedure; Mann-Whitney test for all comparisons). (B) nVNS, administered 30 and 47 min after MCAO for 2 min each, reduced SD frequency after VNS initiation as well. The latency to the first SD, SD amplitude, and velocity remained unchanged (n = 8 rats undergoing nVNS vs. n = 8 rats undergoing a sham VNS procedure; Mann-Whitney test for all comparisons). (C) Relative changes in cerebral blood flow (CBF) in peri-infarct cortex, measured using laser speckle contrast imaging, showed no significant effects of either iVNS or nVNS (n = 8 rats in each group; Mann-Whitney test for both comparisons).

As expected [12], multiple SDs occurred spontaneously in the 4-hour measurement time after pMCAO. In rats subjected to iVNS, initiated 30 min after MCAO onset and administered every 5 min for 1 h, there was a significant reduction of SD frequency (Fig 2A) as well as a significant increase of the latency to the first SD (Fig 2A) in the time window subsequent to the stimulation compared to a sham VNS procedure. The cumulative area under the curve of all SDs in the 4-h window showed a nonsignificant decrease (Fig 2A), whereas SD amplitude and propagation velocity remained unchanged (Fig 2A).

We also determined the effect of nVNS, which was delivered 30 min and 47 min after pMCAO onset. In these animals, SD frequency was also significantly reduced (Fig 2B), while the latency, amplitude and propagation velocity remained unchanged in the time window subsequent to the stimulation compared to a sham VNS procedure (Fig 2B).

To rule out that these effects on SD frequency were related to CBF changes induced by VNS, we analyzed cortical perfusion continuously during pMCAO using laser speckle contrast imaging, and detected no significant effects of iVNS or nVNS on cortical CBF in that time frame (Fig 2C). Likewise, no difference in SD frequency was detectable in the 30-min time window preceding VNS stimulation (iVNS: n = 1.13 ± 0.3 vs. n = 0.75 ± 0.16; nVNS: n = 1.13 ± 0.13 vs. n = 0.88 ± 0.13; p > 0.05, Mann-Whitney test for both comparisons). Moreover, SD frequency was similar in both sham groups (iVNS, 1.15 ± 0.29 SDs/h; nVNS, 0.81 ± 0.11 SDs/h; p = 0.41, Mann-Whitney test), indicating that manual manipulation of the vagus nerve by the silicone tube in the iVNS sham group did not affect this parameter.

### Physiological parameters remain stable during iVNS and nVNS

**Fig 3:**
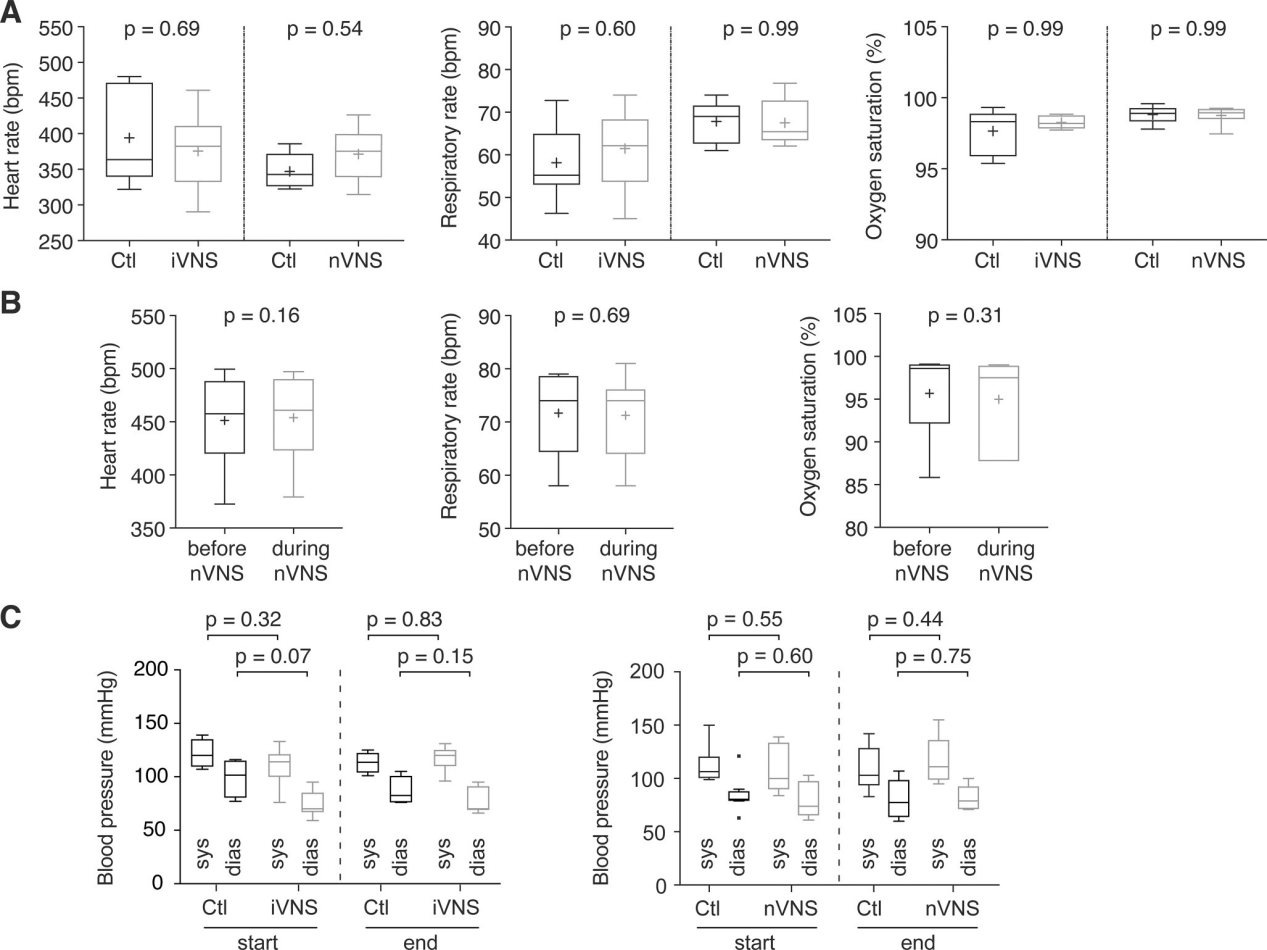
Physiological parameters remain stable under VNS. (A) Heart rate, respiratory rate and oxygen saturation remained stable during the 4-hour time window after pMCAO induction in rats treated with iVNS or nVNS (n = 8 rats in all groups; Mann-Whitney test for all comparisons). (B) Heart rate, respiratory rate (n = 6 rats in each group) and oxygen saturation (n = 7 rats in each group) remained stable when measured immediately before and during nVNS application (Mann-Whitney test for all comparisons). (C) Systolic (sys) and diastolic (dias) blood pressure, measured at the initiation and end of pMCAO, were similar in rats treated with iVNS or nVNS and rats undergoing a sham VNS procedure (n = 8 control rats vs. n = 5 VNS-treated rats; Mann-Whitney test for all comparisons).

We also determined pulse rate, breathing rate and oxygen saturation throughout the 4 h duration of pMCAO in rats receiving iVNS or nVNS compared to animals receiving a sham VNS procedure during MCAO. These measurements showed that VNS had no significant effects on these parameters in that time span (Fig 3A). We also analyzed these parameters at time points immediately before and during nVNS, and again detected no changes compared to rats undergoing a sham VNS procedure (Fig 3B). Moreover, we determined systolic and diastolic blood pressure at the start and endpoint of pMCAO, and detected no significant differences between the groups (Fig 3C). Together, these data indicate that invasive or non-invasive stimulation of the left vagal nerve has no peri-procedural detrimental effects on cardiovascular parameters in this stroke model.

### nVNS reduces cortical infarct volume

**Fig 4:**
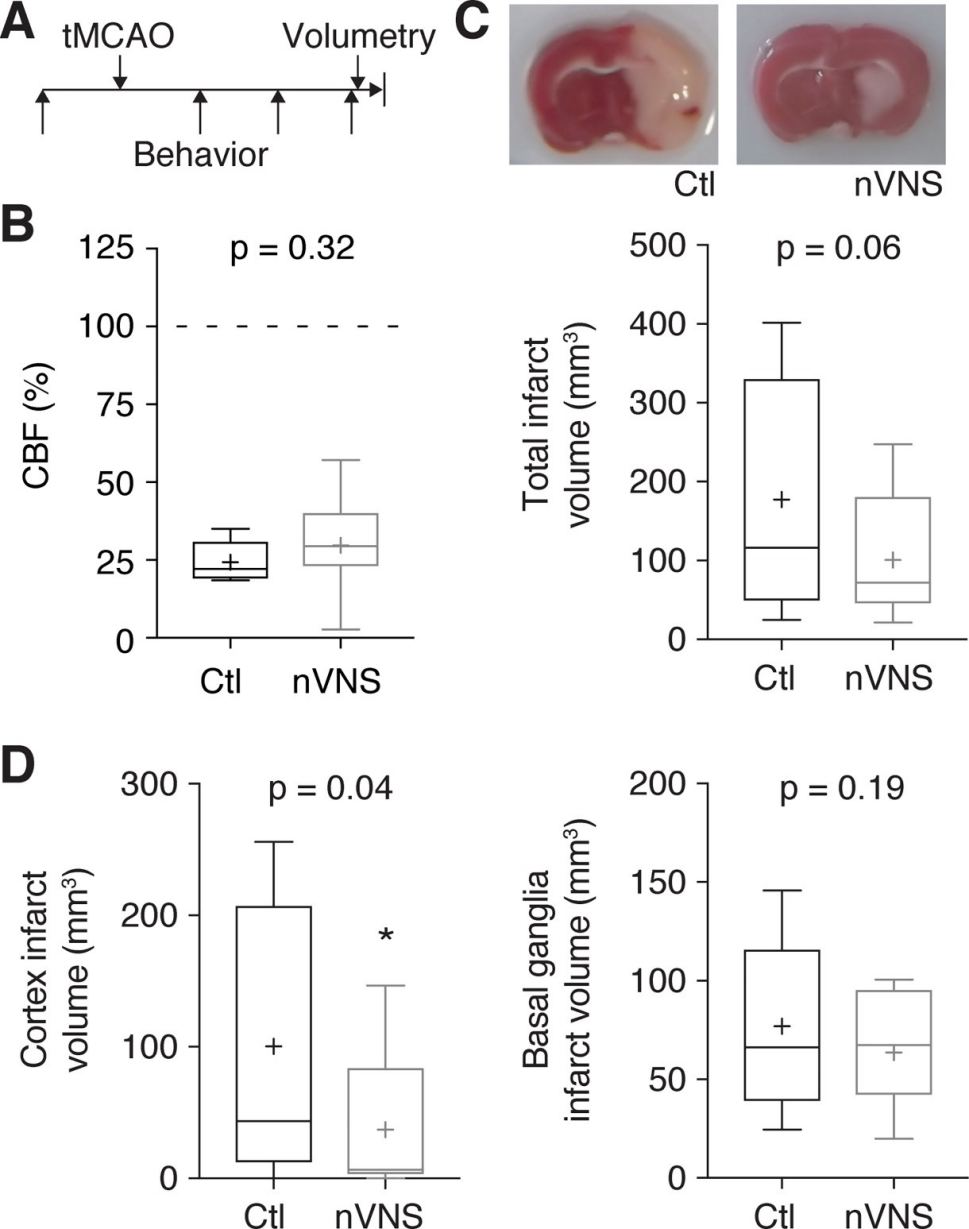
nVNS reduces cortical stroke volume. (A) Transient middle cerebral artery occlusion (tMCAO) was induced for 60 min. Sensorimotor function assessed before as well as 1, 2, and 3 days after tMCAO, followed by post-mortem infarct volumetry. (B) Cerebral blood flow (CBF) relative to baseline, measured through the thinned skull, verified successful MCAO induction and showed no differences between rats treated with nVNS and rats undergoing a sham nVNS procedure (n = 10 control vs. n = 11 VNS-treated rats; unpaired t-test). (C) nVNS led to a non-significant reduction of total infarct volume (n = 11 rats in each group; unpaired t-test; images show representative examples). (D) Separate analysis of cortical and subcortical stroke volume revealed a significant reduction of infarct volume in the cortex by nVNS, whereas infarct volume in the basal ganglia remained unchanged (n = 11 rats in each group; unpaired t-test for both comparisons).

To determine if the reduction of SD frequency induced by VNS in our model results in changes in infarct volume, we compared nVNS to a sham VNS procedure in rats subjected to transient (60 min) MCAO (tMCAO; Fig 4A). We chose tMCAO as opposed to permanent occlusion to replicate our findings in another model as recommended by the STAIR guidelines [21], and as tMCAO may better reflect the majority of large-vessel occlusion strokes in human patients. Because we did not implant cranial windows in these groups, successful MCAO induction in these animals was measured by laser Doppler flowmetry through the thinned skull during tMCAO (Fig 4B). The differences in CBF values measured by laser Doppler flowmetry to those obtained by laser-speckle imaging (Fig 2C) are within the range observed by other studies [22], although we cannot exclude that absolute CBF values differed between pMCAO and tMCAO.

Histological infarct volume determined 72 h after tMCAO revealed a non-significant trend towards smaller infarct sizes in rats subjected to nVNS (Fig 4C). However, when we separately analyzed cortical and subcortical infarct volume–based on the premise that SDs predominantly occur in cortical areas [15]–we found that cortical infarct volume was significantly reduced in the nVNS group, but remained unchanged in subcortical areas (Fig 4D).

### Sensorimotor outcome after VNS

**Fig 5:**
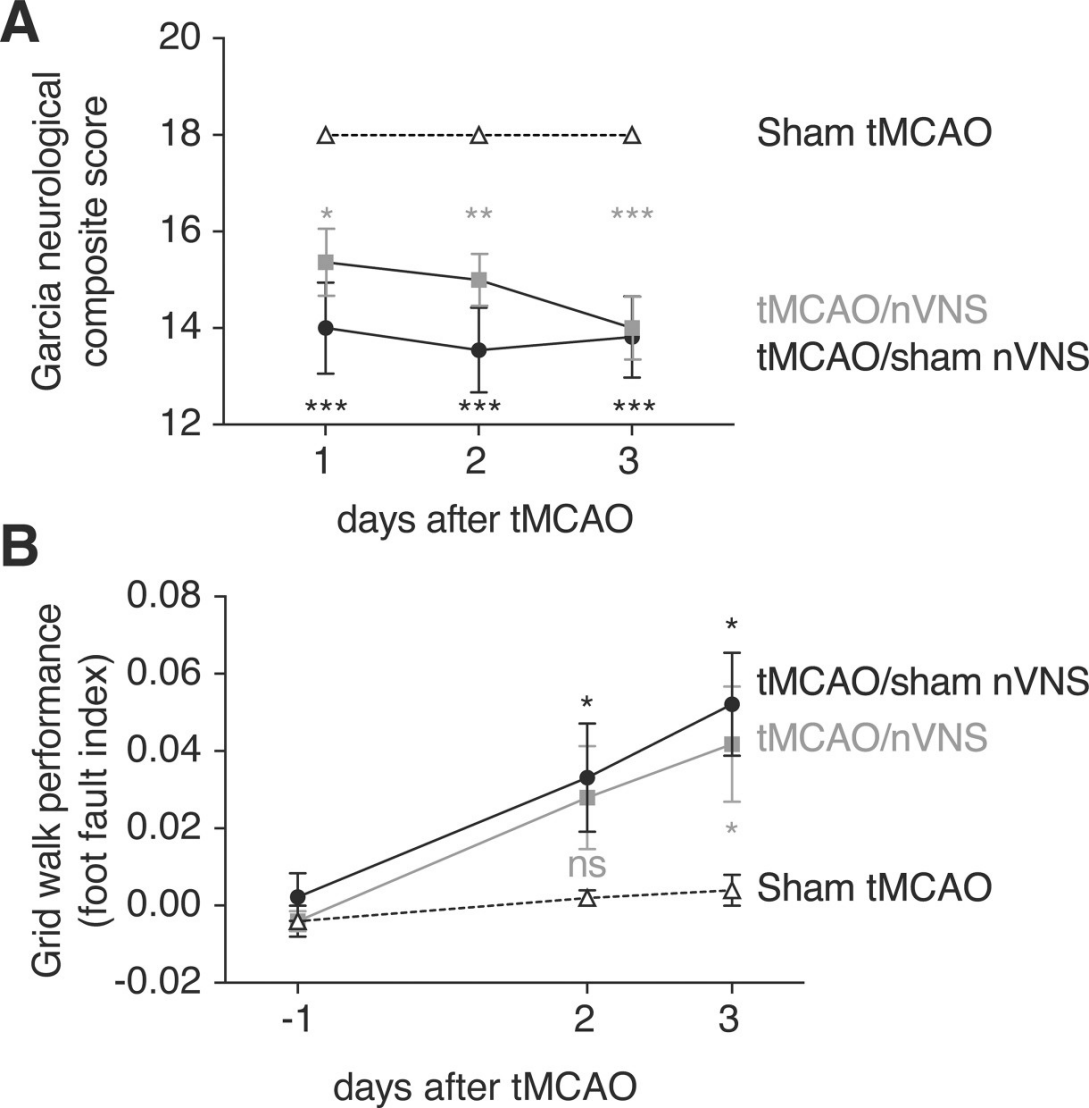
Effects of nVNS on sensorimotor function after focal ischemia. (A) Rats after tMCAO and nVNS and rats after tMCAO and a sham nVNS procedure were compared to rats undergoing MCAO sham surgery using the Garcia neuroscore. Both tMCAO groups showed significantly worse sensorimotor function compared to the sham group (n = 11 rats in each group; two-way repeated measures ANOVA followed by Dunnett’s multiple comparisons test; * p < 0.05, ** p < 0.01, *** p < 0.0001). (B) When motor function of the contralateral forelimb was assessed using the grid walk test, motor performance in rats treated with VNS after tMCAO was similar to rats undergoing sham MCAO on day 2 after surgery, but significantly different in rats having received MCAO and a sham VNS procedure. However, on day 3 after MCAO, both post-MCAO groups performed significantly worse compared to control rats (Sham tMCAO and tMCAO/nVNS, n = 10 rats each; tMCAO/sham nVNS, n = 9 rats; two-way repeated measures ANOVA followed by Dunnett’s multiple comparisons test; * p < 0.05).

As part of the prespecified study protocol, the groups–rats undergoing tMCAO with or without nVNS, and sham-operated control animals–were also tested for sensorimotor deficits using the Garcia neuroscore as well as the grid walk test. As expected, rats undergoing sham tMCAO procedures showed a stable performance in both tests, whereas rats undergoing tMCAO without nVNS showed a significant decrease in sensorimotor function in both tests (Fig 5A and 5B). No difference was observed in the Garcia test in rats after tMCAO together with nVNS (Fig 5A).

At the day 2 time point in the grid walk test, rats having received tMCAO together with nVNS performed similarly as the group undergoing sham MCAO, whereas rats having received tMCAO without VNS performed worse (Fig 5B). However, this difference was not detectable at the 3-day time point, where both tMCAO groups–with and without nVNS–performed worse than the sham group (Fig 5B).

## Discussion

In this preclinical study, we have tested the effect of non-invasive or invasive stimulation of the left cervical branch of the vagus nerve on SD burden in a rat model of focal ischemic stroke. We have found that both stimulation paradigms led to a pronounced reduction of SD frequency in this model. Although this effect was milder than the SD reduction seen in studies using knockout strategies to genetically interfere with critical components of the SD signaling cascade [12], its effect size on SD frequency is comparable to ketamine, whose efficacy has been demonstrated in preclinical and clinical studies [23,24]. Moreover, VNS is easier to administer and control than ketamine, and can be used outside of intensive care units. In addition, physiological parameters including heart rate and blood pressure remained stable in the present study as well as in earlier VNS studies [1,6,7].

Although this study was designed to test for a translationally meaningful efficacy of VNS on SDs, and not to elucidate the molecular and cellular pathways responsible for this effect, it is tempting to speculate about the underlying mechanisms. First, VNS leads to an activation of several brainstem and cerebellar nuclei [25], some of which have strong effects on SD threshold [26]. Second, VNS triggers the release of neurotransmitters such as norepinephrine and serotonin from brainstem nuclei [27–29], which attenuate the propensity for SDs [30,31]. Third, VNS reduces the release of glutamate as well as several pro-inflammatory cytokines [32–34], all of which promote the development of SDs [12,35,36]. Finally, VNS reduced blood-brain barrier disruption in a stroke model [6], which by itself may inhibit SDs [37].

Of note, the reduction of SD frequency was associated with a decrease of cortical stroke volume in our model, whereas basal ganglia stroke volume remained unchanged. Although previous studies only reported total infarct volume [1–3,6], the reported magnitude of stroke volume reduction was similar to the cortical infarct volume reduction observed in our study. Whereas other mechanisms, including the ones mentioned above, likely also contribute to infarct volume reduction, it is interesting to note that SDs mostly occur in cortical areas [8], implicating SD reduction as a major clinically relevant mechanism in stroke therapy by VNS. On the other hand, the beneficial effects on sensorimotor outcome were marginal in our model. In retrospect, this is perhaps not unexpected given the predominantly cortical reduction in infarct volume observed here, which should leave the damaged motor pathways passing through subcortical regions largely unaffected. Hence, it will also be important to utilize tests that assess cortical functions in subsequent studies. In this regard, it is interesting to note that VNS has shown positive effects on cognitive functions after focal ischemia in a preclinical study [5].

The effects of iVNS and nVNS were similar in our study. We did not measure total energy delivered to the vagal nerve during both paradigms, but assuming that each pulse triggers one action potential in both paradigms, the total number of evoked vagus nerve firings per hour were comparable (iVNS, 9,000; nVNS, 12,000), suggesting similar efficacies. Given that nVNS appears safe and can easily be administered in clinical routine, it will also be important to test more stimulations for longer duration, longer delay between stroke onset and stimulation as well combinatorial studies with approved interventions such as intravenous thrombolysis or mechanical thrombectomy.

Finally, spreading depolarizations are not only relevant for the pathophysiology of stroke, but also occur in other conditions such as migraine, subarachnoid hemorrhage and traumatic brain injury [15]. Hence, our study as well as a previous study demonstrating that normoxic spreading depolarizations are also inhibited by VNS [7] collectively suggest that VNS may represent an attractive strategy to reduce detrimental spreading depolarization waves in acute brain disorders.

## Supporting information

S1 Dataset Original data. (XLSX)
